# Viability of Glioblastoma Cells and Fibroblasts in the Presence of Imidazole-Containing Compounds

**DOI:** 10.3390/ijms23105834

**Published:** 2022-05-23

**Authors:** Elisabeth Christiane Seidel, Claudia Birkemeyer, Rainer Baran-Schmidt, Jürgen Meixensberger, Henry Oppermann, Frank Gaunitz

**Affiliations:** 1 Klinik und Poliklinik für Neurochirurgie, Universitätsklinikum Leipzig, 04103 Leipzig, Germany; christiane.seidel@medizin.uni-leipzig.de (C.E.S.); rainer.baran-schmidt@medizin.uni-leipzig.de (R.B.-S.); juergen.meixensberger@medizin.uni-leipzig.de (J.M.); henry.oppermann@medizin.uni-leipzig.de (H.O.); 2 Klinik und Poliklinik für Neurologie, Universitätsklinikum Leipzig, 04103 Leipzig, Germany; 3 Institut für Analytische Chemie, Universität Leipzig, 04103 Leipzig, Germany; birkemeyer@chemie.uni-leipzig.de; 4 Institut für Humangenetik, Universitätsklinikum Leipzig, 04103 Leipzig, Germany

**Keywords:** carnosine, glioblastoma, fibroblasts, imidazole-containing compounds, cell viability, high-performance liquid chromatography coupled to mass spectrometry

## Abstract

The naturally occurring dipeptide carnosine (β-alanyl-*L*-histidine) specifically attenuates tumor growth. Here, we ask whether other small imidazole-containing compounds also affect the viability of tumor cells without affecting non-malignant cells and whether the formation of histamine is involved. Patient-derived fibroblasts and glioblastoma cells were treated with carnosine, *L*-alanyl-*L*-histidine (LA-LH), β-alanyl-*L*-alanine, *L*-histidine, histamine, imidazole, β-alanine, and *L*-alanine. Cell viability was assessed by cell-based assays and microscopy. The intracellular release of *L*-histidine and formation of histamine was investigated by high-performance liquid chromatography coupled to mass spectrometry. Carnosine and LA-LH inhibited tumor cell growth with minor effects on fibroblasts, and *L*-histidine, histamine, and imidazole affected viability in both cell types. Compounds without the imidazole moiety did not diminish viability. In the presence of LA-LH but not in the presence of carnosine, a significant rise in intracellular amounts of histidine was detected in all cells. The formation of histamine was not detectable in the presence of carnosine, LA-LH, or histidine. In conclusion, the imidazole moiety of carnosine contributes to its anti-neoplastic effect, which is also seen in the presence of histidine and LA-LH. Despite the fact that histamine has a strong effect on cell viability, the formation of histamine is not responsible for the effects on the cell viability of carnosine, LA-LH, and histidine.

## 1. Introduction

With 3.23 new cases per 100,000 inhabitants in the United States, glioblastoma (GBM) is the most frequent malignant tumor of the human brain [1]. GBM is an astrocytic tumor that is classified, according to the World Health Organization (WHO), with the highest WHO grade IV. Despite the best possible treatment, which consists of maximal safe resection of the tumor, radiotherapy, and adjuvant chemotherapy with temozolomide, the 5-year overall survival of GBM patients is only 7.2% [1]. Furthermore, the effectiveness of this therapy is highly dependent on the genetic properties of the tumor, namely, the methylation status of the *O*-6-methylguanine-DNA methyltransferase (MGMT) promotor [2]. In view of the poor prognosis and missing alternatives to standard therapy, there is ongoing research for new treatment strategies and drugs that could improve the corresponding outcome.

In recent years, we and others have demonstrated that the naturally occurring dipeptide *L*-carnosine (β-alanyl-*L*-histidine; note that throughout the text, “carnosine” refers to “*L*-carnosine”), which was originally discovered more than 120 years ago [3], may be a potential anti-neoplastic drug for different types of cancer in general and also for glioblastoma in particular (for reviews, see [4,5]). As carnosine is rapidly degraded in human plasma due to the presence of serum carnosinase, it has long been thought that its use as a systemically administered drug may be limited. Although there is now evidence that carnosine can escape from degradation by its uptake into erythrocytes [6], other compounds with higher stability may be considered as alternatives. Therefore, it is reasonable to study and better understand carnosine’s mode of action on tumor cell viability, which also requires an understanding of the bioactive characteristics of the molecule. In view of the observation that *L*-histidine is able to mimic carnosine’s anti-neoplastic effect [7], we asked whether other small imidazole-containing compounds are also able to mimic carnosine’s effect, whether the β-alanyl moiety is required, and whether the effects of other compounds are indeed comparable to those of carnosine. In addition, we also wanted to know whether the formation of histamine from *L*-histidine is involved in the anti-neoplastic effect.

## 2. Results

### 2.1. Viability of Glioblastoma Cells and Patient-Derived Fibroblasts in the Presence of Imidazole-Containing Compounds

**Figure 1:**
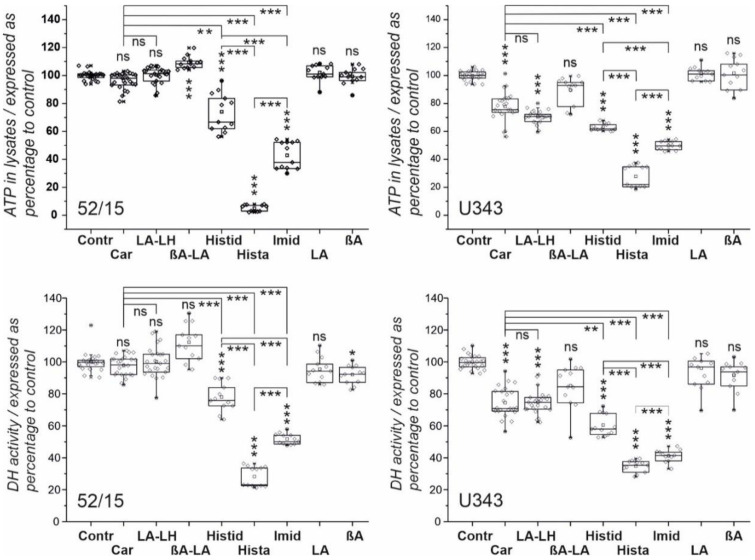
Viability of fibroblasts from culture 52/15 (**left**) and glioblastoma cells from the U343 line (**right**) after treatment with different compounds. Cells were treated for 48 h with carnosine (Car), *L*-alanyl-*L*-histidine (LA-LH), β-alanyl-*L*-alanine (βA-LA), *L*-histidine (Histid), histamine (Hista), imidazole (Imid), *L*-alanine (LA), and β-alanine (βA) (all 50 mM) or vehicle control (Contr) for 48 h. Cell viability was measured by determining ATP in cell lysates (upper panels) and dehydrogenase activity (DH) in living cells (lower panels). Results are presented as box plots. Statistical analysis was performed using a one-way ANOVA. The level of significance between different compounds is indicated by horizontal lines and compared to Contr above the boxes: *: *p* < 0.05; **: *p* < 0.005; ***: *p* < 0.0005; ns: not significant.

In the first series of experiments, we investigated the effect of imidazole-containing compounds on cell viability using five patient-derived fibroblast cell cultures (13/16, 90/15, 69/15, 60/15, 52/15) and four glioblastoma cell lines (U87, T98G, U87, G55T2). The cells were incubated for 48 h in the presence of carnosine, *L*-alanyl-*L*-histidine (LA-LH), β-alanyl-*L*-alanyl (βA-LA), *L*-histidine, histamine, imidazole, *L*-alanine, and β-alanine (all 50 mM), and cell viability was determined measuring the amount of ATP in cell lysates and dehydrogenase (DH) activity in living cells. The data were compared to untreated control cells. The results of experiments with fibroblasts from culture 52/15 and cells from the glioblastoma cell line U343 are presented in Figure 1 (all other experiments are presented in detail in Supplement S1 with Figure S1a for ATP in cell lysates and Figure S1b for dehydrogenase activity).

The summary of all data obtained is shown in Table 1.

In summary, as revealed by both assays, carnosine significantly reduced viability in all glioblastoma cells to at least 80% (with the exception of measuring DH in T98G). As shown in Table 1, fibroblasts also responded with a small reduction of viability in the presence of carnosine in almost all cells (maximal reduction to 90%), which was, in all cases, lower than that observed in GBM cell lines. Using LA-LH, we could also detect a significant reduction of viability in glioblastoma cells, as determined by both assays, whereas the viability of fibroblasts was not affected by the compound. Overall, carnosine and LA-LH exerted a comparable impact on glioblastoma cells, with differences regarding the various cell lines. Histidine reduced glioblastoma cell viability in a stronger manner than carnosine and LA-LH and significantly affected the viability of fibroblasts. In contrast, the non-imidazolyl-containing dipeptide βA-LA did not reduce GBM or fibroblast viability. Histamine strongly reduced fibroblast viability and glioblastoma cell viability without a significant difference between both cell types. Comparing the effect of histamine with histidine, a significantly stronger reduction by histamine was detected in fibroblasts but not in glioblastoma cells. Imidazole also strongly reduced the viability of fibroblasts and glioblastoma cells without discriminating between the cell types. In comparison to imidazole, histamine strongly reduced the cell viability of fibroblasts and exerted a comparable effect on glioblastoma cells.

### 2.2. Necrosis and Morphological Changes of GBM Cells and Patient-Derived Fibroblasts after Treatment with Different Imidazole-Containing Compounds

In the preceding section, we analyzed the viability of GBM cells and fibroblasts by measuring ATP in cell lysates and dehydrogenase activity in living cells after treatment with different compounds. These experiments indicated that carnosine and LA-LH more clearly reduced the investigated parameters in GBM cells than in fibroblasts; histidine and imidazole also appeared to affect GBM cells more severely than fibroblasts, and histamine similarly affected fibroblasts and tumor cells. Nonetheless, only carnosine and LA-LH appeared to have no effect on the viability of fibroblasts, although they obviously inhibited the production of ATP and dehydrogenase activity in GBM cells in general to below 80%.

**Figure 2:**
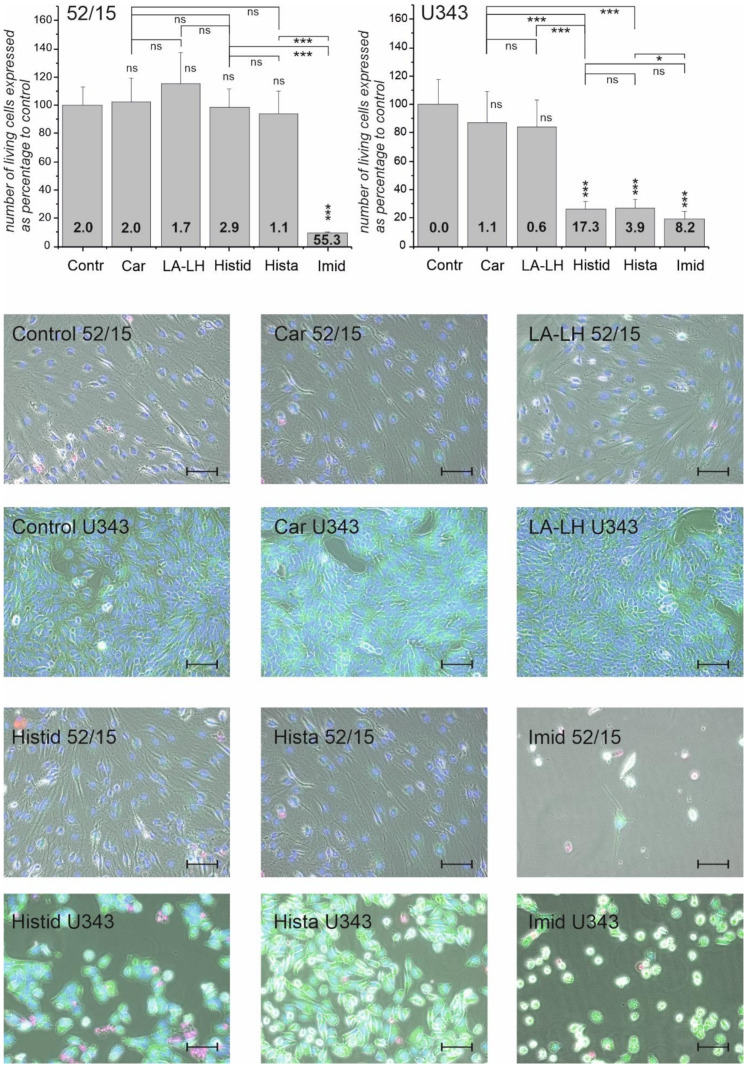
Microscopic analysis of fibroblasts (52/15) and glioblastoma cells (U343) in the presence of different compounds. Cells were treated for 48 h with carnosine (Car), *L*-alanyl-*L*-histidine (LA-LH), β-alanyl-*L*-alanine (βA-LA), *L*-histidine (Histid), histamine (Hista), imidazole (Imid), *L*-alanine (LA), and β-alanine (βA) (all 50 mM) or vehicle control (Contr) for 48 h. Then, microscopic images after staining with Hoechst 33343 (nuclei, blue), propidium iodide (dead cells, red), and Calcein-AM (living cells, green) were compared (representative images are presented as overlays in the lower panels, which also include an image obtained by phase contrast). In order to determine the number of living cells using ImageJ, the total number of nuclei was determined, subtracting the nuclei of dead cells (bars in upper panel; note: determination of living cells from images of cells positive for Calcein-AM staining was not performed because of high errors due to technical reasons). The ratio of dead cells to living cells is presented by bold numbers in the bars in the upper panel. Statistical analysis was performed using a one-way ANOVA. The level of significance between different compounds is indicated by horizontal lines and compared to Contr above the bars: *: *p* < 0.05; ***: *p* < 0.0005; ns: not significant. (Size bars: 100 µm).

In order to get a more detailed picture of the effect of the compounds on GBM cells and fibroblasts, we investigated the subsequent morphological changes after treatment with the selected compounds, and we determined cell numbers and necrotic cells by propidium iodide staining. In addition, we also stained living cells using Calcein-AM. For this experiment, cells from the four glioblastoma cell lines (G55T2, T98G, U87, and U343) and from fibroblast cultures (90/15, 69/15, 60/15, and 52/15) were exposed to our compounds (each 50 mM) for 48 h. Then, staining with Hoechst 33343, Calcein-AM, and propidium iodide was performed, and the effects were monitored by phase contrast and fluorescence microscopy. Figure 2 presents the result of the experiment and its analysis, along with representative pictures of cells from the U343 line and from fibroblast culture 52/15. A summary of all data obtained is presented in Table 2.

Pictures from experiments with other cell lines and fibroblast cultures are presented in Supplement S2 with microscopic images for 53/15 (Figure S2a); 60/15 (Figure S2b); 69/15 (Figure S2c); 90/15 (Figure S2d); G55T2 (Figure S2e); T98G (Figure S2f); U87 (Figure S2g); U343 (Figure S2h) and the statistical analysis (Figure S2i). In addition, a summary of the observations from all cells is given in Table 2.

In summary, the strongest reduction of the number of living cells was achieved in the presence of imidazole, which did not significantly discriminate between fibroblasts or glioblastoma cells, indicating the generally high toxicity of this compound for both cell types. In some cases (e.g., U87), the loss of cells was so high that the calculation of the ratio between dead and living cells was not useful (“nd” in Table 2). Histidine and histamine, on the other hand, affected fibroblasts and glioblastoma cells significantly differently, with many more dead cells in the latter. Compared to histidine and histamine, carnosine and LA-LH seemed to be less toxic but, in most cases, exhibited a stronger effect on glioblastoma cells than on fibroblasts. Comparing the cell counts presented in Table 2 to the effects on physiological parameters (Table 1), it seems to be likely that the effects of the different compounds on cells may differ with regard to their mechanisms of action on energy metabolism.

### 2.3. The Release of L-Histidine from L-Alanyl-L-Histidine Does Not Result in the Formation of Histamine

**Figure 3:**
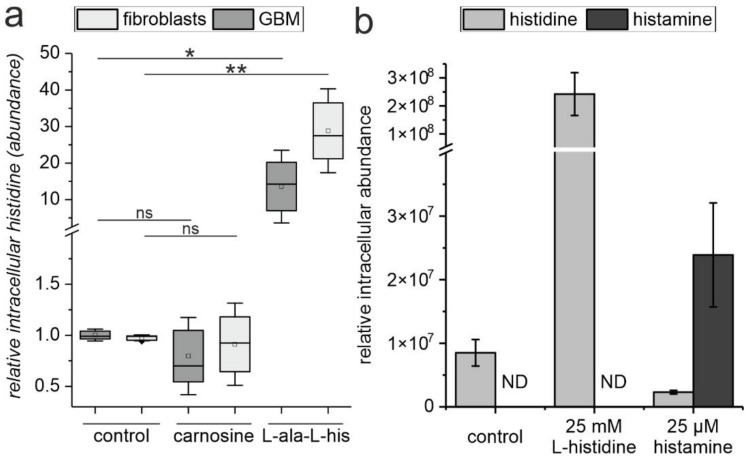
Release of histidine from carnosine and LA-LH and formation of histamine from histidine in fibroblasts and glioblastoma cells. (**a**) Four different glioblastoma cell lines and five different fibroblast cultures were treated with carnosine or *L*-alanyl-*L*-histidine (LA-LH) (both 50 mM) or vehicle control for 48 h. Afterwards, metabolites were extracted and intracellular histidine was determined by LC–MS. Results are presented as box plots that were obtained from the median of the replicates of each cell culture. (**b**) T98G cells were treated with 25 mM L-histidine, 25 µM histamine, or vehicle control for 48 h. Afterwards, metabolites were extracted, and intracellular histamine and *L*-histidine were determined by LC–MS. Statistical analysis was performed using a one-way ANOVA with the Games–Howell post hoc test. The level of significance is indicated as: *: *p* < 0.05; **: *p* < 0.005; not significant (ns): *p* > 0.05. ND: not detected.

Next, we investigated whether different amounts of *L*-histidine are released from carnosine and LA-LH and whether significant intracellular amounts of histamine can be formed from intracellular *L*-histidine. Figure 3a presents the intracellular amounts of *L*-histidine when cells are incubated in the presence of carnosine or LA-LH, respectively (both 50 mM), as determined in fibroblast cell cultures (*n* = 5) and glioblastoma cell lines (*n* = 4). As can be seen, no significant increase of intracellular *L*-histidine compared to untreated control cells was observed in cells incubated in the presence of carnosine. In contrast, incubation in the presence of LA-LH resulted in a significant increase in intracellular *L*-histidine. In order to detect a possible formation of histamine, we incubated cells from the GBM line T98G directly in the presence of *L*-histidine (25 mM) and histamine (25 µM) and determined the amount of both compounds in the cells. As can be seen in Figure 3b, we could not detect histamine in cells incubated in the presence of *L*-histidine, although we observed a steep rise in the intracellular amount of histamine when added to the medium. In addition, we did not detect histamine or its degradation products in cells exposed to carnosine, LA-LH, or *L*-histidine, whereas we observed the formation of *N*-methylhistamine in cells cultivated in the presence of histamine.

## 3. Discussion

Several decades ago, the anti-neoplastic effect of carnosine was first described in vivo by Nagai and Suda [8]. Later, this observation was confirmed in vivo and in vitro by several groups and for different types of cancer, such as gastric carcinoma [9], colon carcinoma [10], cervical carcinoma [11], and glioblastoma [12]. Just recently, we demonstrated that L-histidine, one amino acid of the dipeptide, reduces GBM cell viability even more potently than carnosine [13]. Therefore, we wondered whether L-histidine itself or other histidine-containing compounds would also discriminate between malignant GBM cells and non-malignant fibroblasts, as demonstrated for carnosine [14,15]. Here, we observed that carnosine significantly reduced cell viability in GBM cells, whereas there was no significant effect observed in fibroblasts with regard to the amount of ATP in cell lysates and only a small but significant effect with regard to dehydrogenase activity. A comparable discrimination between tumor and non-tumor cells was also observed after the application of LA-LH. Although L-histidine did significantly reduce the viability of fibroblasts, the effect was stronger in GBM cells but significantly different between GBM cells and fibroblasts only with regard to dehydrogenase activity.

As L-histidine also has anti-neoplastic effects on GBM cells, this observation raises the question of whether *L*-histidine could be used as a therapeutic agent instead of carnosine. First of all, at the concentrations employed in our experiments (50 mM), the amino acid obviously also affects fibroblasts. Comparable observations have been made by Rauen et al., who detected that in cultivated liver cells, *L*-histidine at a concentration of 76 mM had a ~2.5 higher toxicity compared to carnosine at a concentration of 198 mM [16]. As *L*-histidine is a proteinogenic amino acid, it could also be assumed that orally ingested *L*-histidine is taken up by other cells and may not reach the tumor. Orally applied carnosine, on the other hand, is rapidly degraded by serum carnosinase [17] and may, therefore, be limited in delivering its histidine moiety to cancer cells. In this regard, it has to be noted that we recently demonstrated that carnosine can escape from degradation by uptake into erythrocytes [6], explaining the observation that the dipeptide can be detected in the urine of volunteers up to 5 h after oral ingestion [18]. In addition, there are several reports of the therapeutic effects of orally ingested carnosine that point towards the delivery of intact carnosine, especially to the brain [19,20,21].

Given the fact that LA-LH has an anti-neoplastic effect comparable to carnosine, being able to discriminate between tumor and non-tumor cells, the other question is whether this compound or other imidazole-containing dipeptides could be a useful alternative to carnosine. This question cannot be answered yet, but it is interesting to note that our experiments demonstrate that LA-LH is intracellularly more rapidly degraded to its amino acid constituents than carnosine (Figure 3). Therefore, future experiments should investigate whether the release of *L*-histidine results in a more rapid loss of the bioactive imidazolyl moiety in the cells and whether more stable *L*-histidine-containing dipeptides could be an alternative to carnosine or LA-LH. More complex synthetic compounds derived from imidazole have already been discussed as potential anti-cancer drugs (for a review, see [22]). In fact, some of the more complex imidazole-derived compounds have already entered the clinics with regard to a number of diseases (for a review, see [23]), but one has to take into account that synthetic drugs require intensive testing before being used in therapy. On the other hand, carnosine is a naturally occurring compound that has already been used in a number of studies with human patients, and, together with its constituent β-alanine, it has a high acceptance as a food supplement for athletes [24]. At this point, it is also interesting to note that carnosine, instead of negatively affecting non-malignant cells, has protective effects on normal cells and has been discussed as a neuroprotector, especially in various pathological brain conditions [25]. Imidazole itself has also been discussed as a potential drug for the treatment of colon cancer [26]. However, although Long and Wang used low concentrations in their culture experiments with colon carcinoma cells (up to 36 µM), the high toxicity towards fibroblasts, as seen in our experiments, should be taken into account when considering the use of imidazole as an anti-cancer drug.

Another question addressed by the presented experiments is whether histamine obtained by decarboxylation of *L*-histidine could be responsible for carnosine’s and L-histidine’s anti-neoplastic effect. This notion has been discussed by others [27] and could have been deduced by the fact that histamine has a very strong effect on viability (Figure 1). However, we now rule out this possibility by the observation that we could not detect the formation of histamine after the exposure of GBM cells to carnosine, LA-LH, or *L*-histidine. In addition, we also did not detect *N*-methylhistamine, a degradation product of histamine, in cells exposed to carnosine, LA-LH, or L-histidine, which were detectable in cells exposed to histamine. At this point, it should also be noted that *AOC1* (gene encoding diamine oxidase (EC 1.4.3.22), which is responsible for the conversion of *L*-histidine to histamine) is almost never present in GBM cells and normal brain tissue (transcripts per million transcripts ~2), as revealed by in silico analysis using data from the TCGA Research Networks (https://www.cancer.gov/tcga; accessed on 24 December 2022) and the GTex database (https://gtexportal.org/home/; accessed on 24 December 2022) using GEPIA (Gene Expression Profiling Interactive Analysis; http://gepia.cancer-pku.cn/; accessed on 24 December 2022 [28]). In addition, it should be noted that the neuroprotective properties of carnosine are also independent of its metabolization via the *L*-histidine-histamine pathway [29].

In conclusion, our experiments demonstrate the importance of the *L*-histidine moiety of carnosine for its anti-neoplastic effect. Although the molecular mechanisms by which this moiety exerts its anti-neoplastic effect have to be revealed in detail, there is evidence that imidazolyl-containing compounds are able to inhibit mitochondrial ATP production [30]. In addition, it has been shown that they can induce cell cycle arrest [31], most likely by binding the imidazolyl moiety to DNA [32]. Despite the observation that the imidazolyl moiety contributes to the anti-neoplastic effect, it is important to note that we recently demonstrated that carnosine’s influence on tumor cell viability is accompanied by an influence on the pentose phosphate pathway through its interaction with the glycolytic intermediates glyceraldehyde-3-phosphate and dihydroxyacetone phosphate. Therefore, carnosine may have a broader influence on tumor cells than *L*-histidine alone or other imidazolyl-containing dipeptides [13]. Finally, it would also be interesting to test whether the other compounds used in our study also affect the migration of GBM cells, as demonstrated for carnosine [15]. On the other hand, at least for compounds with high toxicity, such as *L*-histidine, imidazole, or histamine, one certainly needs to test lower concentrations than those used in our present investigation.

## 4. Material and Methods

### 4.1. Reagents

If not stated otherwise, all chemicals were purchased from Sigma-Aldrich (Taufkirchen, Germany), Merck (Darmstadt, Germany), or Carl Roth (Karlsruhe, Germany). Carnosine was kindly provided by Flamma s.p.a. (Chignolo d’Isola, Italy); *L*-alanyl-*L*-histidine and β-alanyl-*L*-alanine were purchased from Bachem (Bubendorf, Switzerland).

### 4.2. Cell Lines and Fibroblast Cultures

The glioblastoma cell line G55T2 was obtained from Sigma (Taufkirchen, Germany), the cell lines U87 and T98G from the ATCC (Manassas, VA, USA), and the U343 line from the German Collection of Microorganisms and Cell Cultures (Braunschweig, Germany). All cells were genotyped (Genolytic GmbH, Leipzig, Germany), and their identities were confirmed.

For cultivation, cells were propagated in 250 mL culture flasks (Sarstedt AG & Co., Nümbrecht, Germany) using 10 mL of standard culture medium (DMEM/4.5 g/L glucose, without pyruvate (Life Technologies, Darmstadt, Germany) supplemented with 10% fetal bovine serum (FBS superior, Biochrom, Berlin, Germany), 2 mM GlutaMax (Life Technologies), and penicillin–streptomycin (Life Technologies)) at 37 °C and 5% CO_2_ in humidified air in an incubator.

Fibroblast cultures were established as described previously [15]. Briefly, freshly removed galea tissue was washed with PBS (phosphate-buffered saline) and minced with a scalpel blade. After mincing, small tissue pieces were transferred to a 25 cm^2^ culture flask (TPP, Trasadingen, Switzerland) sprinkled with AmnioMax complete medium (Gibco, Darmstadt, Germany). Tissue pieces were incubated for 30 min at room temperature, and finally, 1 mL AmnioMax complete medium was added. Incubation was then performed at 37 °C, with 5% CO_2_ and humidified air in an incubator. Medium was changed after 72 h. As soon as a confluent layer was obtained, cells were removed from culture flasks by the use of Accutase (PAA, Pasching, Austria) and transferred to 75 cm^2^ culture flasks (TPP). AmnioMax medium with AmnioMax supplement was used for the first 2–3 weeks of cultivation. Thereafter, fibroblasts were cultivated under the same conditions as glioblastoma cells. Galea tissue was obtained during standard surgery performed at the Neurosurgery Department of the University Hospital Leipzig in 2015 and 2016. All patients provided written informed consent according to German law, as confirmed by the local committee (144/08-ek).

### 4.3. Cell Viability Assays

For cell viability assays, cells were counted and seeded into sterile 96-well plates (μClear, Greiner Bio One, Frickenhausen, Germany) at a density of 5000 cells/well in 200 μL standard medium. After 24 h of cultivation (37 °C, 5% CO_2_/95% air), the medium was aspirated and fresh medium containing supplements was added, as indicated in each experiment (100 μL/well), and the cells were incubated for an additional 48 h. Then, the CellTiter-Glo Luminescent Cell Viability Assay (Promega, Mannheim, Germany) was employed to determine viable cells by measuring ATP in cell lysates, and the CellTiter-Blue Cell Viability Assay (Promega) was used to quantify the cell’s dehydrogenase activity in living cells. All assays were carried out according to the manufacturer’s protocols. Luminescence and fluorescence were measured using a SpectraMax M5 multilabel reader (Molecular Devices, Biberach, Germany).

### 4.4. Staining and Determination of Live and Dead Cells

The number of live and dead cells after treatment with different compounds was determined in 12-well plates (TPP). Cells were seeded at a density of 80,000 cells per well in 1 mL of medium. After 24 h, cells received fresh medium with the test compounds. After 48 h in the presence of the compounds, cells were washed with Hanks balanced salt solution (calcium, magnesium, 1 g/L glucose, pH 7.4; Thermo Fisher Scientific, Darmstadt, Germany) before DMEM containing Calcein-AM (2 µM), propidium iodide (1.5 µM), and Hoechst 33343 (2 µM) was added for 1 h. Microscopic pictures were taken using a BZ-X800 microscope (Keyence, Neu-Isenburg, Germany) using phase contrast and fluorescence to identify nuclei (360/460 nm), living cells (470/525 nm), and dead cells (560/630 nm). For the determination of the number of nuclei and of dead cells, 9 images (at 4× magnification) from each well were taken, and ImageJ was used to determine the number of dead cells and nuclei [33]. (Note: we used this approach instead of a FACS analysis, as the detachment of fibroblasts, especially under high toxicity conditions, contributes to additional toxicity.)

### 4.5. Determination of Intracellular L-Histidine

Intracellular amounts of histidine and histamine were determined as described previously [13]. Briefly, cells were seeded at a density of 300,000 cells per well into a 6-well plate in 2 mL of culture medium. After 24-h cultivation, the culture medium was removed and replaced with fresh medium containing specific compounds for each experiment, and cells were incubated for an additional 48 h. Then, cells were washed thrice with 1 mL of ice-cold washing buffer, followed by extraction and by the addition of 400 µL of ice-cold methanol. After 10 min of gentle shaking on ice, extracts were collected in 1.5 mL Eppendorf tubes and wells were rinsed twice in 400 µL distilled high-quality water (Milli-Q). Samples were evaporated to dryness by lyophilization (Martin Christ Gefriertrocknungsanlagen, Osterode, Germany). For derivatization, the freeze-dried extracts were redissolved in 100 µL high-quality water (Milli-Q), and 100 µL 0.5% *ortho*-phthalaldehyde (dissolved in methanol) was added. Derivatization was carried out at 37 °C in a thermomixer for 45 min, followed by the addition of 800 µL 0.1% formic acid in HPLC grade water. The obtained solution (200 µL) was transferred into 250 µL conic glass inserts of 2 mL ND10 vials, followed by high-performance liquid chromatography coupled to mass spectrometry (HPLC–MS). After extraction, the protein of the remaining layer of fixed cells was extracted by the addition of 200 µL lysis buffer (77 mM K_2_HPO_4_, 23 mM KH_2_PO_4_, 0.2% TritonX-100, pH 7.8). Then, the total protein was determined by using the Pierce 660 nm Protein Assay (Thermo Scientific, Braunschweig, Germany).

### 4.6. HPLC–MS Set Up and Data Analysis

An Agilent 1100 series HPLC consisting of a variable wavelength detector, a well plate autosampler, and a binary pump, coupled with a Bruker Esquire 3000 plus electrospray ionization mass spectrometer, was used. The column was a Phenomenex Gemini 5 µ C18 110 Å 150 mm × 2 mm column with a precolumn. The eluent system consisted of two solvents, with eluent A: 0.1% formic acid in acetonitrile and eluent B: 0.1% formic acid in HPLC grade water. Mobile phase flow rate was 0.5 mL/min with the following gradient for separation: 0–10 min 90% B, 90% to 0% B within 15 min, 25–35 min 0% B, 0% to 90% B within 5 min, and 40–47 min 90% B for column equilibration. The mass spectrometer operated in positive mode (target mass: *m*/*z* 300; mass range: *m*/*z* 70–400), and the dry gas temperature was set to 360 °C (flow rate: 11 L/min; 70 psi). Data were analyzed using OpenChrom version 2.0.103.v20150204-1700 [34]. Histidine and histamine were identified by standards, and target masses *m*/*z* 272 (histidine) and 228 (histamine) were used for quantification. If not stated otherwise, the abundance of a metabolite is defined by the peak area determined from the selected ion chromatogram of an experiment, normalized to the total cellular protein (µg). It should be noted that this method does not allow discrimination between *D* and *L* stereoisomers. Thus, when referring to signals obtained by HPLC–MS, only histidine (and not *L*-histidine) is mentioned.

### 4.7. Statistical Analysis and Graphical Representation

Statistical analysis was carried out using SPSS (IBM, Armonk, NY, USA; version: 28.0.0.0 (190)). For multiple comparisons, a one-way ANOVA after testing for normality of distribution (Kolmogorov–Smirnov test) was employed, using a Games–Howell or Bonferroni post hoc test after testing for equality of variances (Levene’s test). Results were considered to be statistically significant at a value of *p* < 0.05. Graphical representations were prepared using OriginPro (2021b; OriginLab Corporation, Northampton, MA, USA) and CorelDraw Graphics Suite 2020 (Corel Corporation, Ottawa, ON, Canada).

## Figures and Tables

**Table 1:** Effect of different compounds on viability of fibroblasts and glioblastoma cells.

	Fibroblasts										Glioblastoma Cells							
Culture	13/16		90/15		69/15		60/15		52/15		U343		T98G		U87		G55T2	
Assay	ATP	DH	ATP	DH	ATP	DH	ATP	DH	ATP	DH	ATP	DH	ATP	DH	ATP	DH	ATP	DH
**Carnosine**																		
**LA-LH**																		
**Histidine**																		
**Imidazole**																		
**Histamine**																		
bAla-L-Ala																		
L-Ala																		
b-Ala																		
**Reduction to control (%):**					**100–90**		**90–80**		**80–70**		**70–60**		**60–50**		**50–40**		**<40**	

The reduction of ATP in cell lysates and dehydrogenase activity (DH) in living cells is color-indicated compared to untreated control cells after 48 h exposure to the compounds. Imidazole-containing compounds are shown in bold. Note: only statistically significant effects are indicated, and white fields indicate no significance.

**Table 2:** Comparison of living cells and the ratio between dead and living cells in fibroblasts and glioblastoma cell cultures under the influence of different compounds.

	Fibroblasts								Glioblastoma							
culture	90/15		69/15		60/15		52/15		U343		T98G		G55T2		U87	
	%	ratio	%	ratio	%	ratio	%	ratio	%	ratio	%	ratio	%	ratio	%	ratio
Car																
LALH																
Histid																
Imid																nd
Hista																
**living cells to control (%)**						**≥ 100**	**<100–90**	**<90–80**	**<80–70**	**<70–60**	**<60–50**	**<50–40**	**<40–30**	**<30–20**	**<20–10**	**<10**
											
**ratio dead/living**							**≤0.5**	**>0.5–1**	**>1–2**	**>2–4**	**>4–8**	**>8–16**	**>16–32**	**>32–64**	**>65**	

The total number of living cells in four fibroblast cultures and four glioblastoma cell lines after treatment with different compounds compared to untreated control cells (in %); the ratios between dead cells and living cells are color-indicated.

## Data Availability

All data used in this study are presented in the manuscript and Supplementary Materials.

## Supplementary Materials

The following are available online at https://www.mdpi.com/article/10.3390/ijms23105834/s1 Supplement S1: Viability of cells from four glioblastomas and from five fibroblast cultures derived from patients, cultivated in the presence of imidazole-containing compounds. Supplement S2: Microscopic analysis of fibroblast cell cultures and glioblastoma cells in the presence of different compounds.
