# Supplementary material for: Viability of Glioblastoma Cells and Fibroblasts in the Presence of Imidazole-Containing Compounds
Source: Int J Mol Sci. 2022 May 23;23(10):5834. doi: 10.3390/ijms23105834 (PMC9146156; doi:10.3390/ijms23105834)
Supplement: Supplementary file 1 [file ijms-23-05834-s001.zip › Supplement 1_revised_for_proof.pdf]

Supplement 1

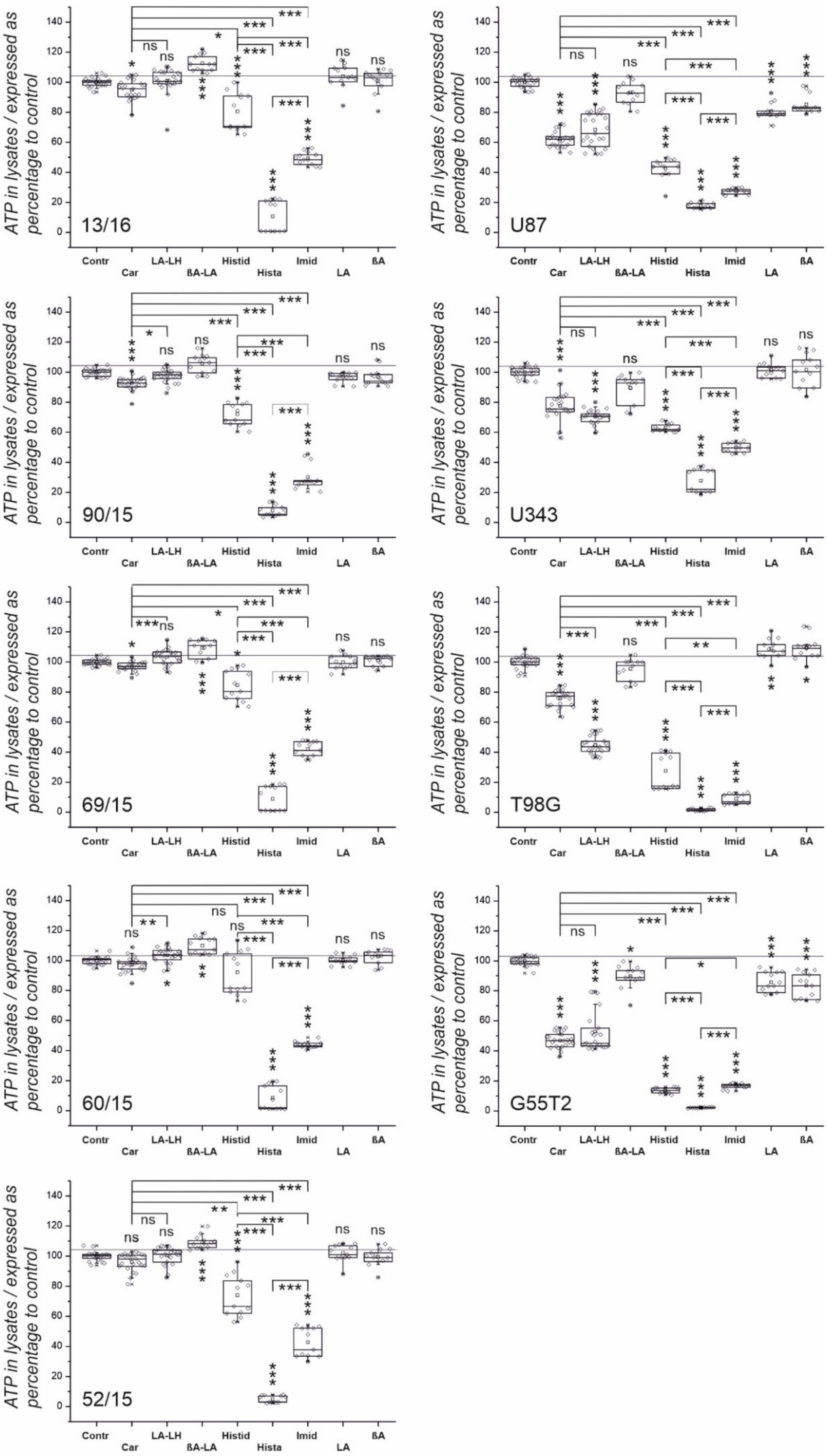

Figure S1.1

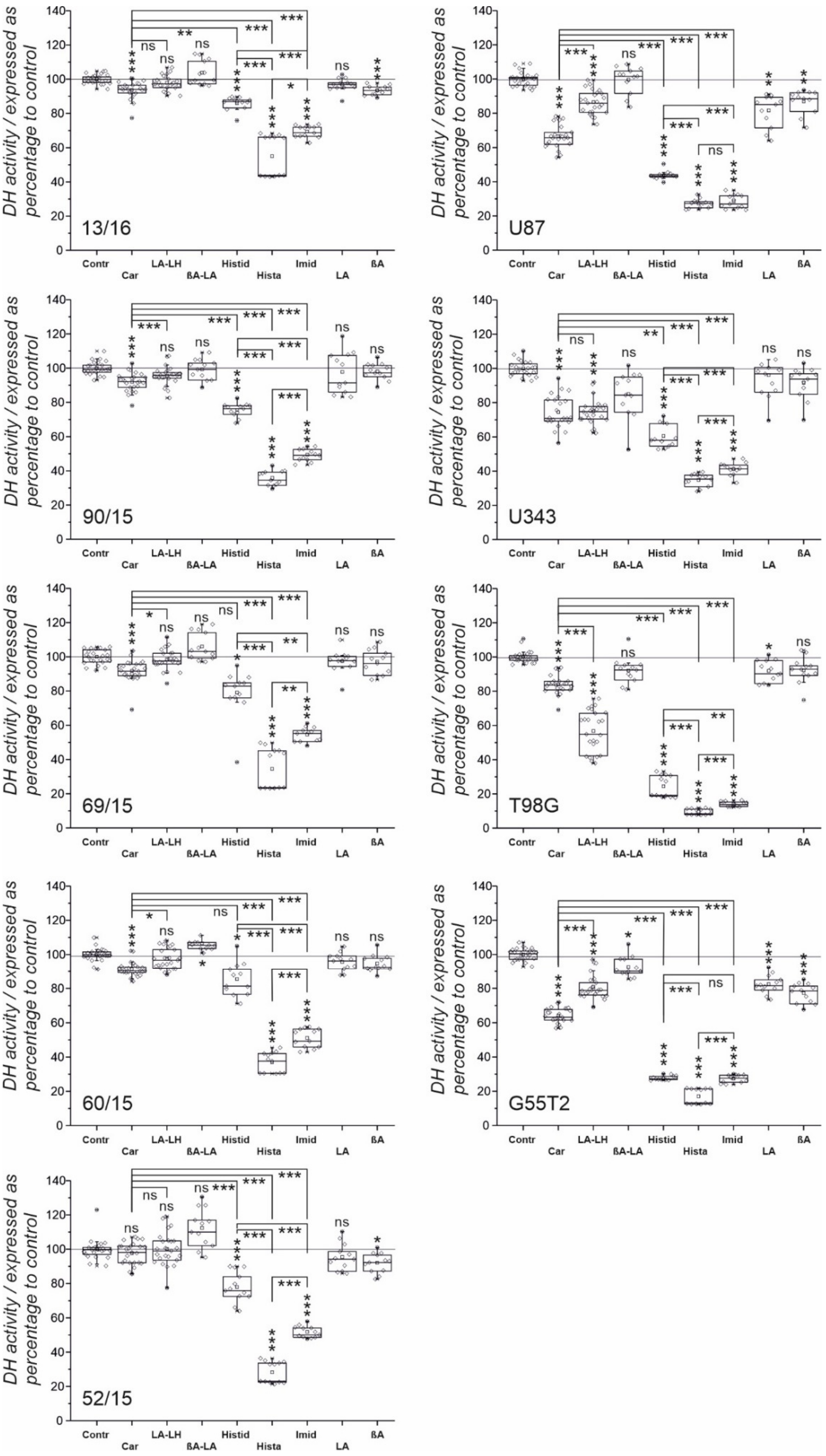

Figure S1.2

Figure S1. Viability of cells from four glioblastoma and from five fibroblasts cultures derived from patients, cultivated in the presence of imidazole-containing compounds. Patient-derived fibroblast cell cultures (13/16, 90/15, 69/15, 60/15, 52/15) and glioblastoma cell lines (U87, T98G, U87, G55T2) were incubated for 48 hours in the presence of carnosine (Car), L-alanyl-L-histidine (LA-LH), -alanyl-L-alanyl (A-LA), L-histidine (Histid), histamine (Hista), imidazole (Imid), L-alanine (LA) and -alanine (A) (all 50 mM). Cell viability was determined measuring the amount of ATP in cell lysates (Fig. S1.1) and dehydrogenase (DH) activity in living cells (Fig. S1.2). Results are presented as box-plots. Statistical analysis was performed using a one-way ANOVA after testing for normality of distribution (Kolmogorov-Smirnov test) and dependent on testing for equality of variances (Levene's test) using a Games-Howell or Bonferroni post hoc test. The level of significance between different compounds is indicated by horizontal lines and compared to Contr above the boxes: *: $p<0.05$; **: $p<0.005$; ***: $p<0.0005$; ns: not significant.
