# Supplementary material for: Viability of Glioblastoma Cells and Fibroblasts in the Presence of Imidazole-Containing Compounds
Source: Int J Mol Sci. 2022 May 23;23(10):5834. doi: 10.3390/ijms23105834 (PMC9146156; doi:10.3390/ijms23105834)
Supplement: Supplementary file 1 [file ijms-23-05834-s001.zip › Supplement 2_revised_for_proof.pdf]

Supplement 2

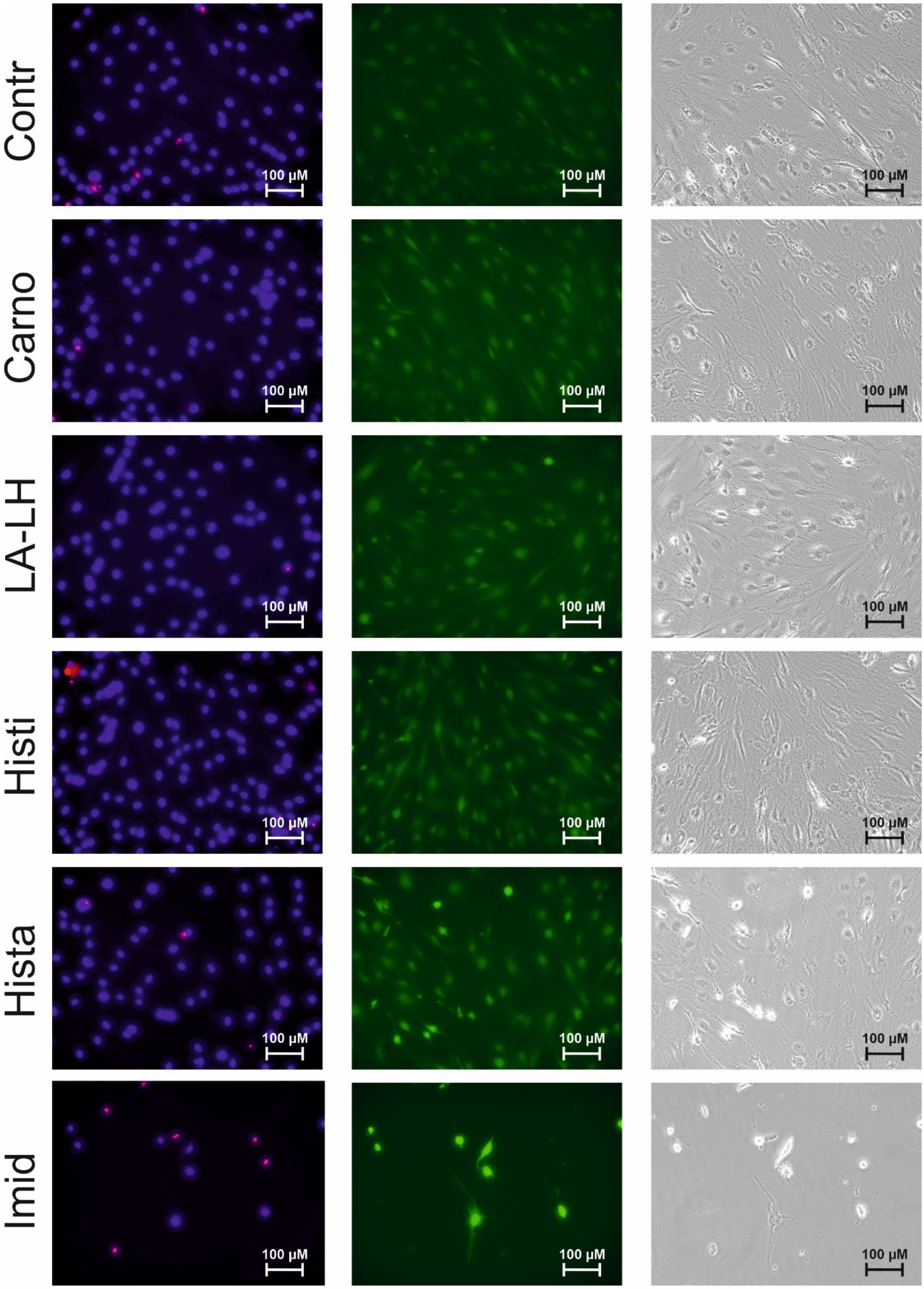

Figure S2.1

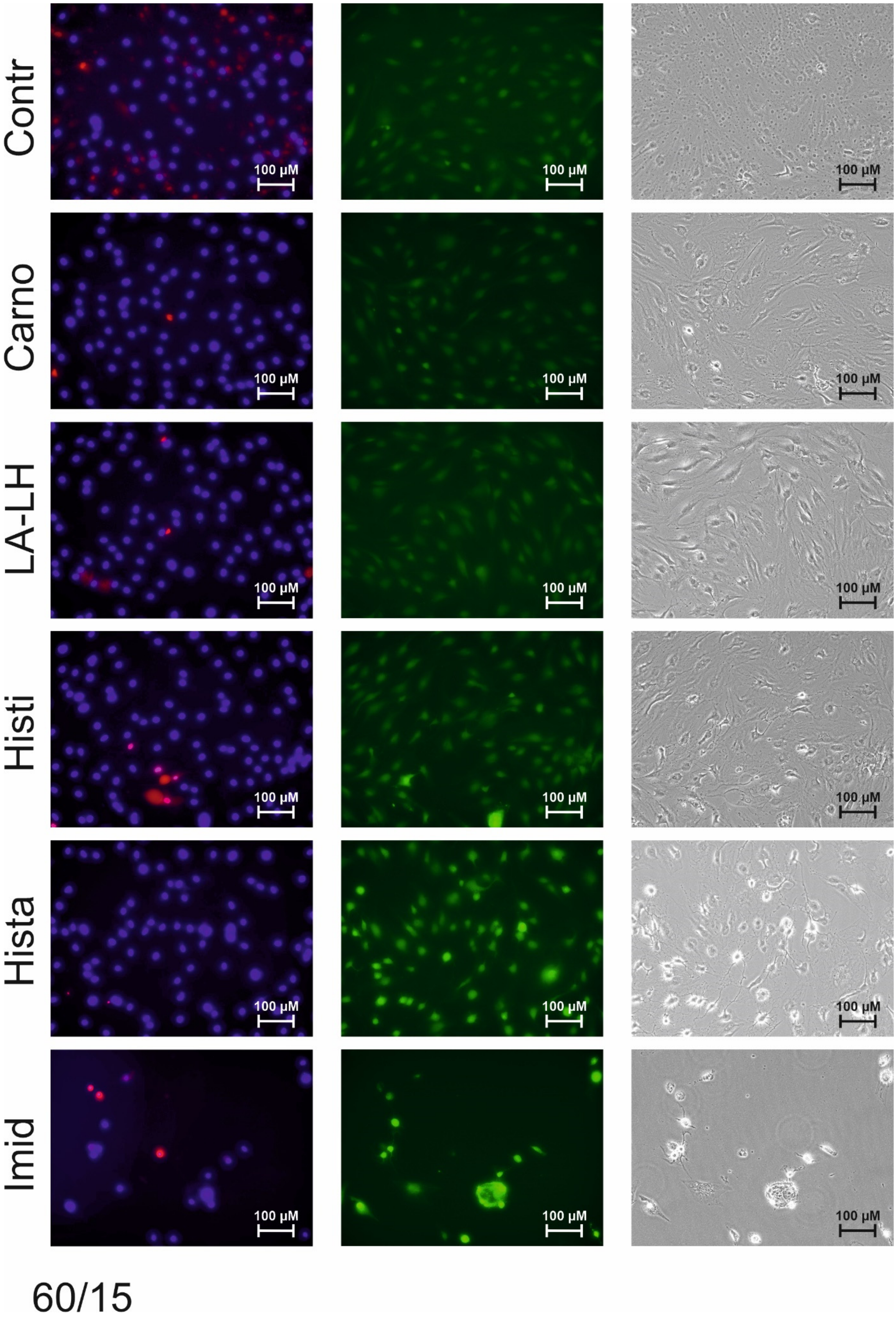

Figure S2.2

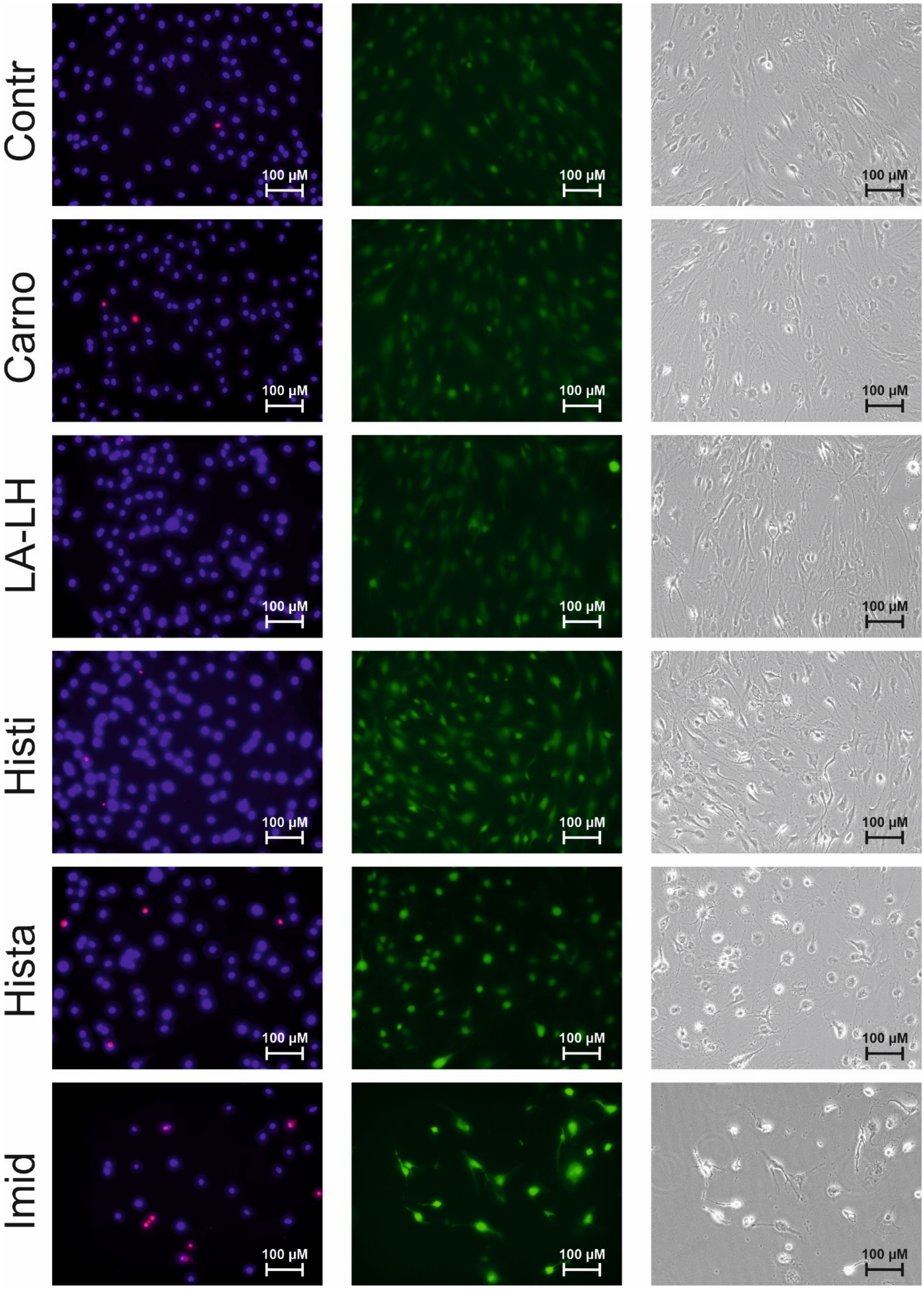

Figure S2.3

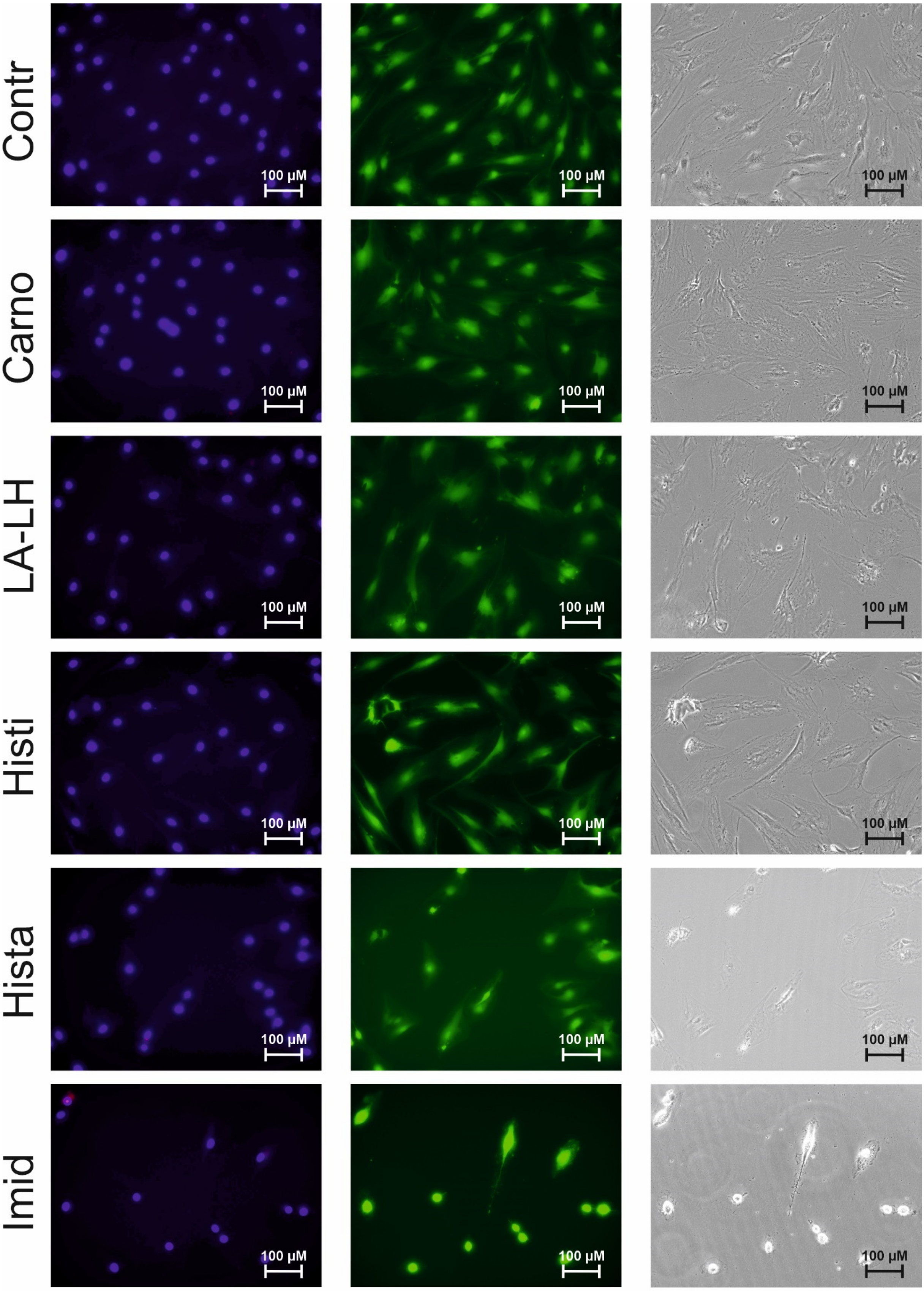

Figure S2.4

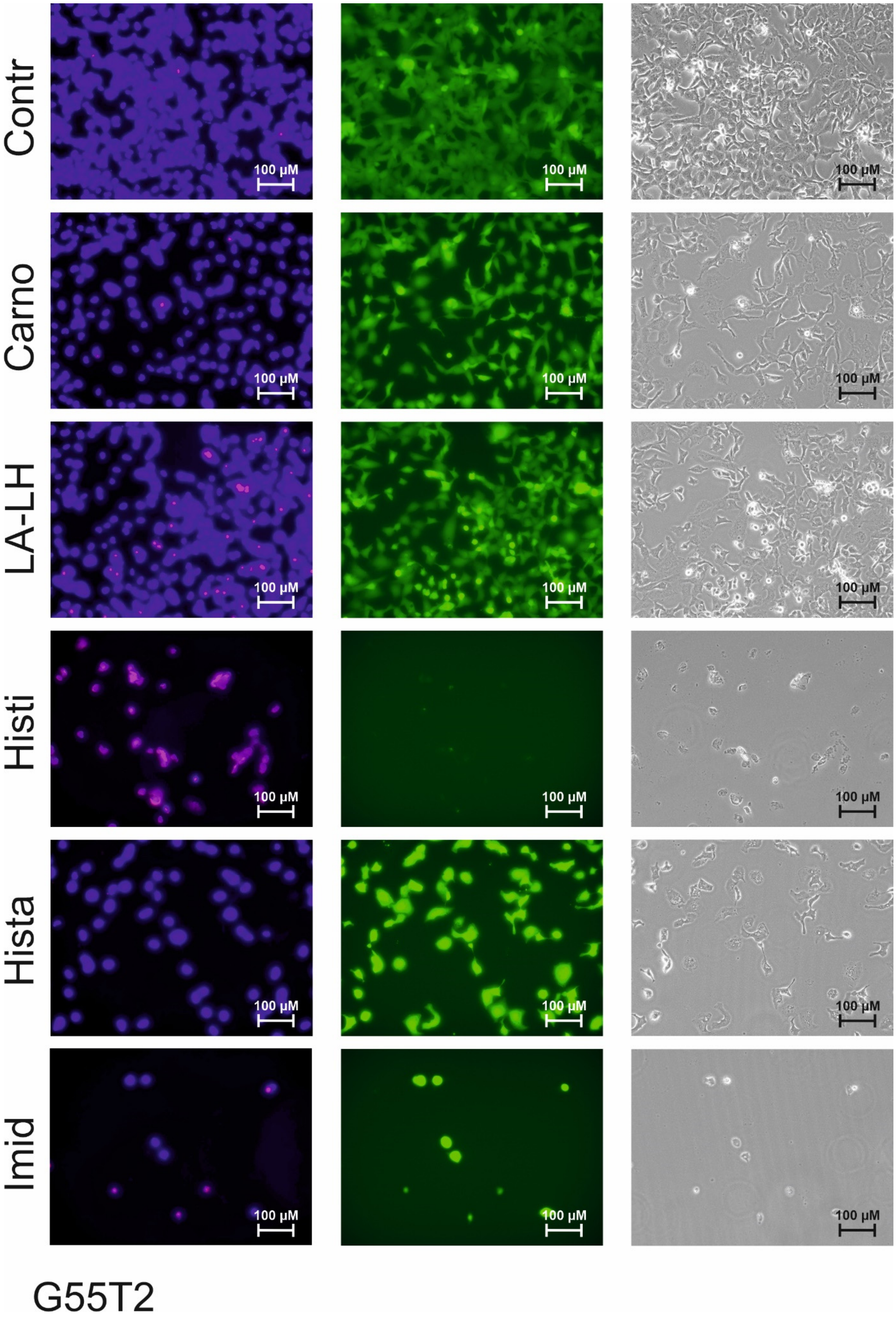

Figure S2.5

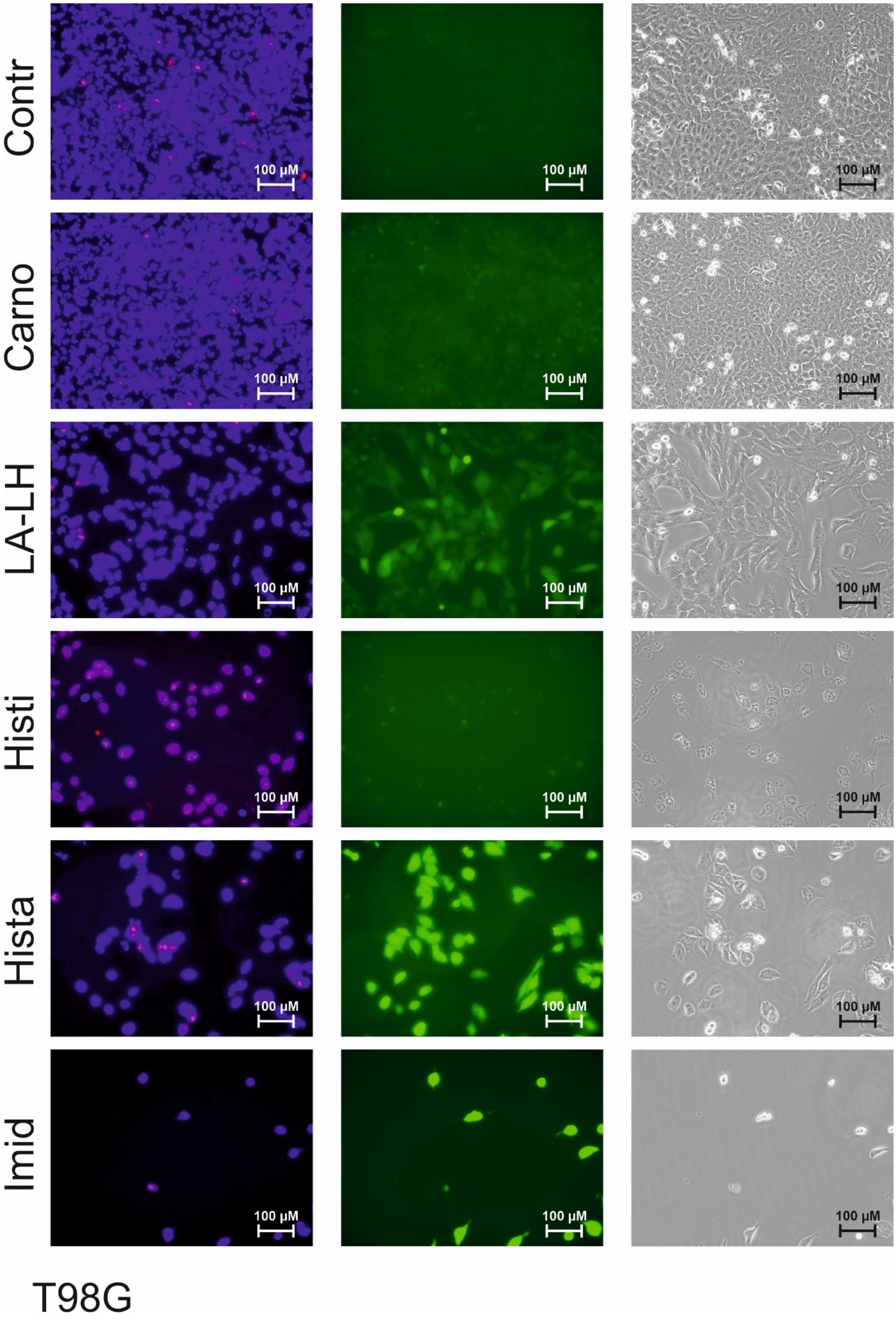

Figure S2.6

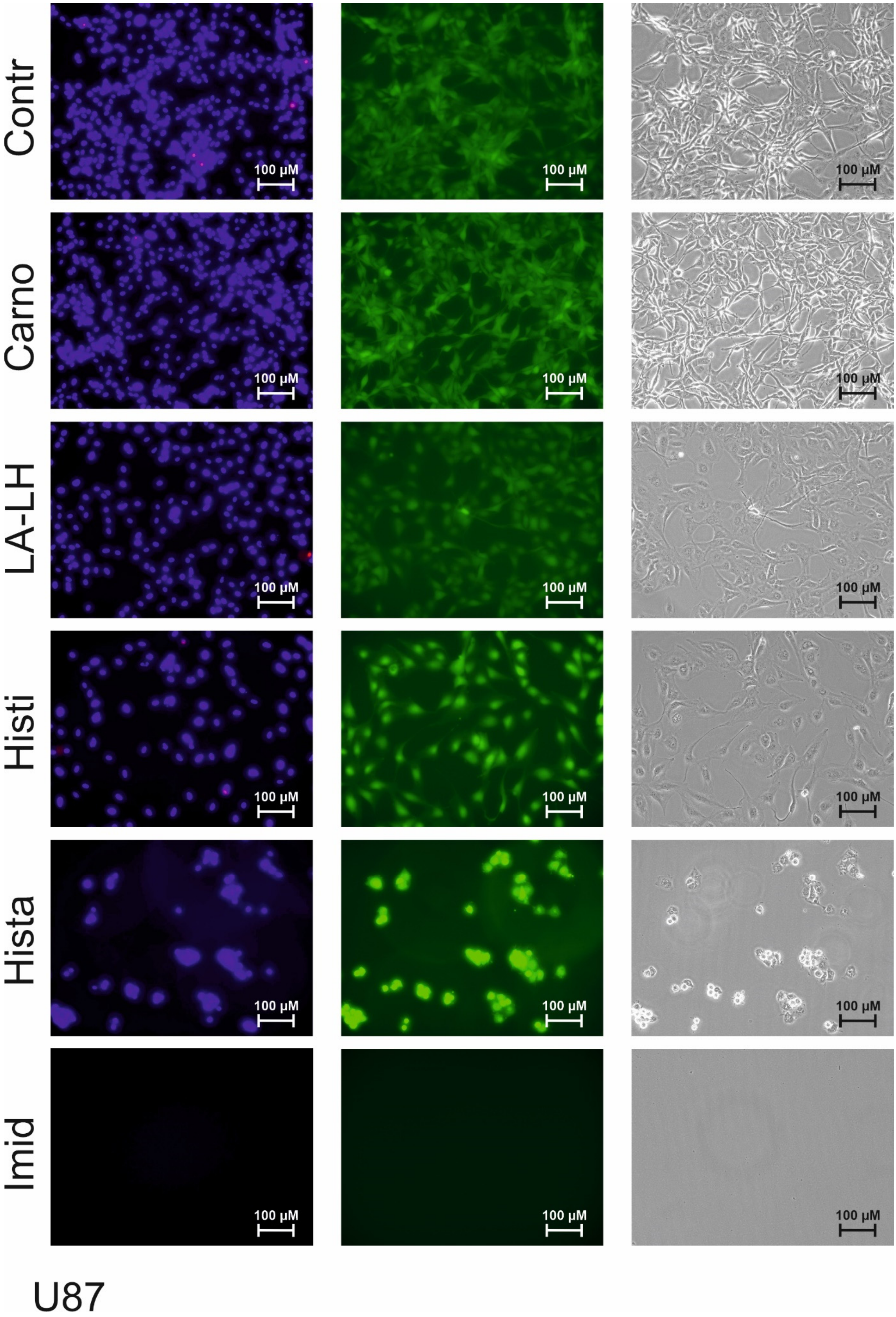

Figure S2.7

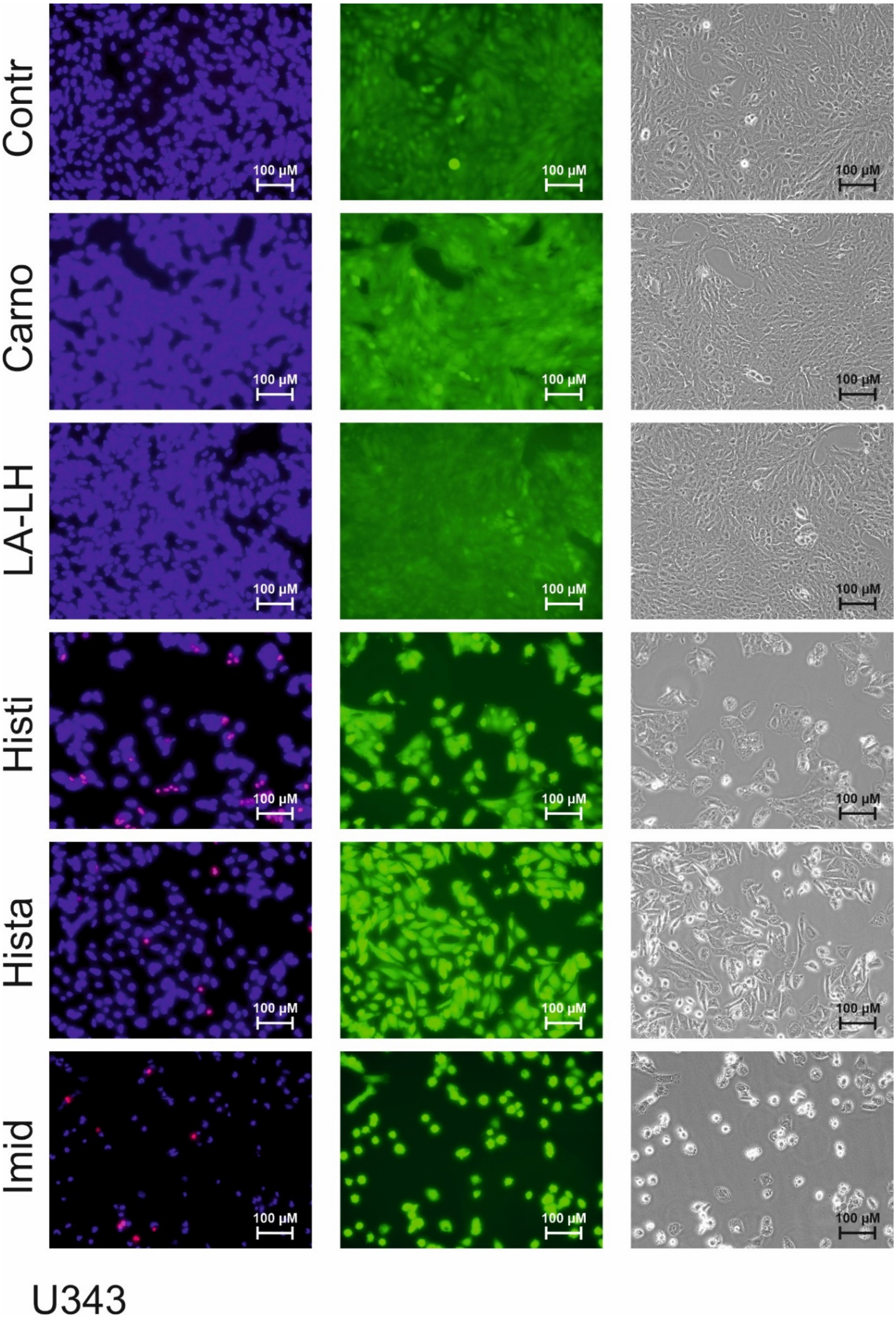

Figure S2.8

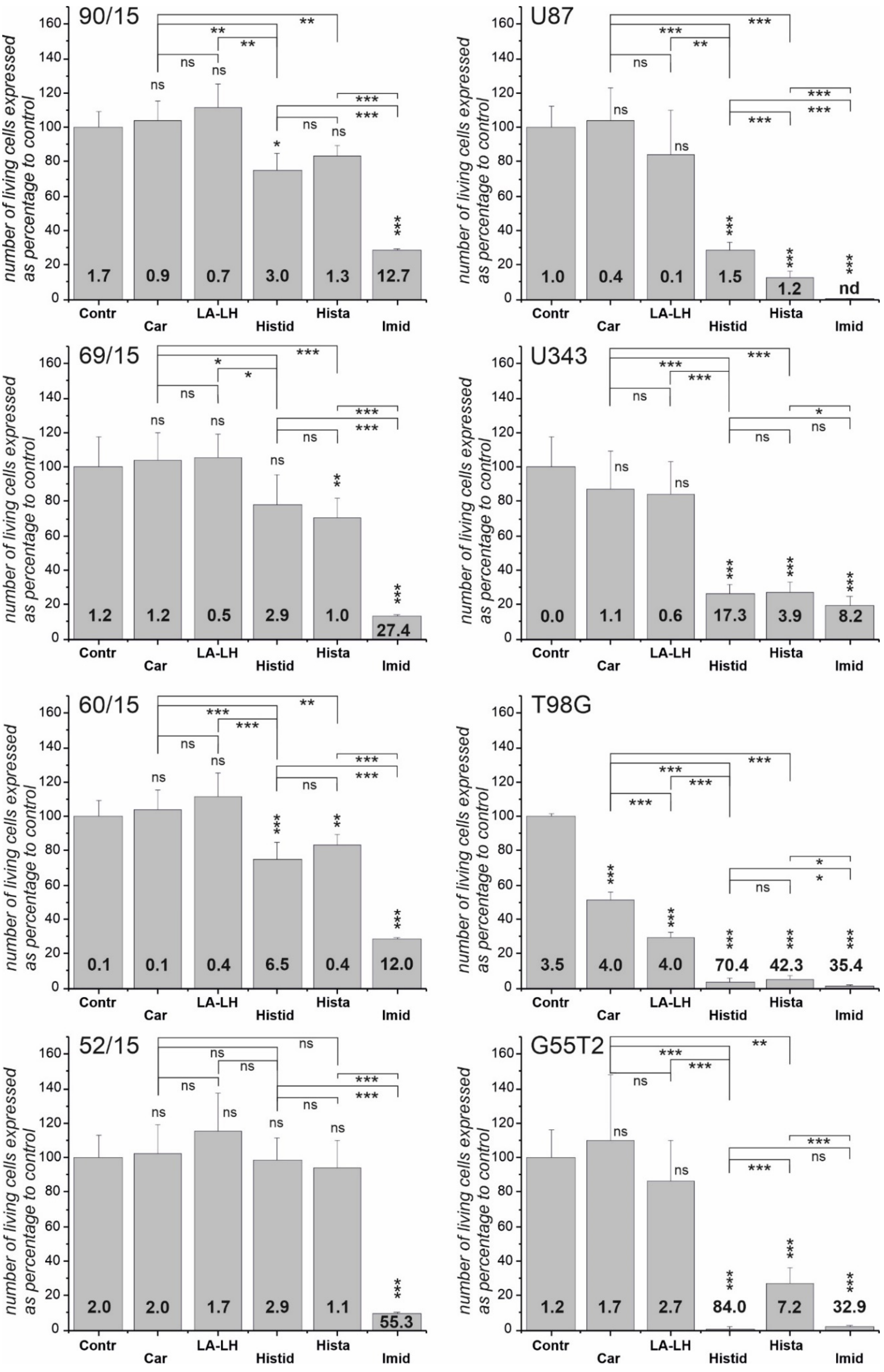
90/15
U87
69/15
U343
60/15
T98G
52/15
G55T2
number of living cells expressed as percentage to control
Contr
Car
LA-LH
Histid
Hista
Imid
1.7 0.9 0.7 3.0 1.3 12.7
1.0 0.4 0.1 1.5 1.2 nd
1.2 1.2 0.5 2.9 1.0 27.4
0.0 1.1 0.6 17.3 3.9 8.2
0.1 0.1 0.4 6.5 0.4 12.0
3.5 4.0 4.0 70.4 42.3 35.4
2.0 2.0 1.7 2.9 1.1 55.3
1.2 1.7 2.7 84.0 7.2 32.9

Figure S2.9

Figure S2. Microscopic analysis of fibroblast cell cultures and glioblastoma cells in the presence of different compounds. Cells were treated for 48 hours with carnosine (Carno), L-alanyl-L-histidine (LA-LH), L-histidine (Histi), histamine (Hista), imidazole (Imid) (all 50 mM) or vehicle control (Contr) for 48 hours. Then, microscopic images after staining with Hoechst 33343 (nuclei, blue), propidium iodide (dead cells, red) and Calcein-AM (living cells, green) was performed. Representative images for all investigated cultures are presented on the following pages (Fig. S2.1: Fibroblasts 52/15; Fig. S2.2: Fibroblasts 60/15; Fig. S2.3: Fibroblasts 69/15; Fig. S2.4: Fibroblasts 90/15; Fig. S2.5: GBM cell line G55T2; Fig. S2.6: GBM cell line T98G; Fig. S2.7: GBM cell line U87; Fig. S2.8: GBM cell line U343). On the left of each panel collection an overlay of nuclei (blue) and of dead cells (red) is shown. The center of each panel collection shows images obtained visualizing Calcein-AM staining of living cells and to the right phase contrast images are shown. Note: in order to compare all images with each other, the same exposition times were used which in some cases results in a blurring image. In Fig. S2.9 box blots are shown that represent the number of living cells under each condition and for all cultures employed (bars) and the ratio of living to dead cells (bold numbers in the bars) as determined by cell counting using ImageJ. Statistical analysis was performed using a one-way ANOVA. The level of significance between different compounds is indicated by horizontal lines and compared to Contr above the bars: *: $p<0.05$; **: $p<0.005$; ***: $p<0.0005$; ns: not significant. (Size bars: 100 µm).
